# *Laminaria japonica* Polysaccharides Improves the Growth Performance and Faecal Digestive Enzyme Activity of Weaned Piglets

**DOI:** 10.3390/vetsci11010011

**Published:** 2023-12-25

**Authors:** Chengwei Wang, Wenning Chen, Yun Xu, Shaomeng Fu, Jiamin Fu, Xiaohong Huang, Junfeng Xiao, Tao Liu, Xianren Jiang

**Affiliations:** 1 College of Life Science, Jiangxi Science and Technology Normal University, Nanchang 330013, China; yung_xy@outlook.com (Y.X.); shaomeng_fu@126.com (S.F.); fjm02608830@outlook.com (J.F.); 2 Key Laboratory of Feed Biotechnology of the Ministry of Agriculture and Rural Affairs, Institute of Feed Research, Chinese Academy of Agricultural Sciences, Beijing 100081, China; chenwn808@126.com; 3 Jiangxi Biotech Vocational College, Nanchang 330200, China; huangxiaohonglove@163.com; 4 Key Laboratory of Swine Nutrition and Feed Science of Fujian Province, Aonong Group, Zhangzhou 363000, China; junfengxiao2023@163.com; 5 Department of Pharmaceutical Sciences, College of Pharmacy, University of Michigan, Ann Arbor, MI 48109-1065, USA; taolius@umich.edu

**Keywords:** amylase, ADG, ADFI, health, lipase

## Abstract

**Simple Summary:**

Weaning is a critical stage in a piglet’s life, and the transition from milk to solid feed after weaning is a challenge to the digestive and metabolic capacity of piglets, resulting in lower feed intake and growth rate as well as a higher incidence of post-weaning diarrhoea. Thus, many functional additives have been used as alternatives to antibiotics to improve the health and welfare of piglets since the ban on the use of antibiotic growth promoters in livestock. Plant-derived natural polysaccharides have great potential to replace antibiotic additives. In the field of natural substances, polysaccharides from seaweed species such as kelp are some of the bioactive molecules that have been investigated as feed supplements with positive effects on animal health parameters, which are important for animal nutrition. Bioactive compounds are recognized as attractive dietary interventions for pigs to improve their intestinal health and reduce the incidence of intestinal diseases. *Laminaria japonica polysaccharides*, as one of them, have prebiotic, anti-microbial, antioxidant, anti-inflammatory and immunomodulatory effects. The aim of this study was to test the hypothesis that the addition of appropriate levels of LJP to the diets of weaned piglets would positively modulate their growth performance, faecal digestive enzyme activity, and serum amino acid content. In this study, we observed that *Laminaria japonica polysaccharides* could improve growth performance and faecal digestive enzyme activities in weaned piglets.

**Abstract:**

The aim of this experiment was to investigate the effect of *Laminaria japonica* polysaccharide (LJP) supplementation at levels of 100, 200, or 400 mg/kg on the growth performance, faecal digestive enzyme activity, and serum biochemistry and amino acids of weaned piglets. One hundred and twenty weaned piglets (Barkshire × Licha Black, 21 days old, 6.13 ± 0.16 kg) were randomly divided into four groups with five replicates of six piglets in each group based on body weight. Piglets were fed with different levels (0, 100, 200, and 400 mg/kg) of LJP for a 21-day trial. On day 21, faecal and blood samples were collected from one piglet per pen. The results showed that the supplementation of the 200 and 400 mg/kg LJP significantly increased average daily gain (ADG) and average daily feed intake (ADFI) compared to the control group (*p* = 0.007; *p* = 0.002), and dietary LJP linearly increased ADG and ADFI (*p* = 0.002; *p* < 0.001). In addition, the supplementation of the 200 and 400 mg/kg LJP significantly increased faecal amylase activity (*p* < 0.001) compared to the control group, and dietary LJP linearly increased faecal amylase and lipase activities (*p* = 0.001; *p* = 0.037). Moreover, dietary LJP at 400 mg/kg increased serum histidine content compared to the other groups (*p* = 0.002), and dietary LJP linearly increased the contents of serum histidine and asparagine in piglets (*p* < 0.001; *p* = 0.046). In conclusion, supplementation of 200 and 400 mg/kg LJP could enhance growth performance and faecal digestive enzyme activity and modulate the serum amino acid content of weaned piglets, potentially contributing to the health of weaned piglets.

## 1. Introduction

Weaning is a critical stage in the life of piglets, and piglets are subjected to psychological, environmental, and nutritional stressors that place significant pressure on piglets. Among them, nutritional stress is the most influential of the weaning stresses. Sow’s milk is the main source of nutrition for piglets during lactation, and the sudden cessation of sow’s milk intake leads to a sudden change in the composition of the diet. This dietary change coupled with the introduction of solid feeds leads to a decrease in the feed intake of weaned piglets [1]. Consequently, inadequate nutrient intake impairs piglets’ digestive capacity with a subsequent decrease in enzyme activity in the gastrointestinal tract [2], affecting the digestion and absorption of nutrients, which may hamper growth and overall health. In addition, the gastrointestinal tract undergoes significant changes during the weaning process. Gut maturation and adaptation to solid feed intake require changes in the composition and function of the gut microbiota. Weaning stress typically disrupts this delicate balance, resulting in a disruption of the balance of gut microbial populations. This disruption can lead to an increased susceptibility to gastrointestinal disorders that manifest as diarrhoea and compromise overall gut health.

*Laminaria japonica* is rich in *Laminaria japonica* polysaccharides (LJPs), which are characterized by their high molecular weight and complex branched structures, featuring a combination of different sugar residues, such as glucose, mannose, fucose, xylose, and glucuronic acid. These structural properties make *Laminaria japonica* polysaccharides exhibit a variety of beneficial biological functions, such as antioxidant [3], inflammatory [4], hypoglycemic [5], hypolipidemic [5], and immune-enhancing [6,7] activities, as well as the regulation of the gut microbiota [8] and other physiological activities. *Laminaria japonica* polysaccharides have a positive effect on improving intestinal health in piglets. A reduction in *Enterobacteria* spp. and an increase in *Lactobacilli* spp. has been shown in the proximal and distal colon of weaned piglets fed with diets containing laminarin [9], with similar results observed by Murphy et al. (2010) [10]. O’Shea et al. (2016) also found that *Laminaria japonica* polysaccharides and fucoidan improved the growth performance of weaned piglets, reduced diarrhoea incidence, and reduced Enterobacteriaceae proximal to the colon [11]. Leonard et al. (2012) found that supplementation with *Laminaria japonica* extracts in the sows’ diet from d 107 of gestation until weaning increased colostrum IgA and IgG concentrations in sows and enhanced immune function and colonic flora in piglets at weaning [12]. Our previous study observed that dietary *Laminaria japonica* polysaccharides at levels of 200 and 400 mg/kg could improve the growth performance and regulate the systemic defence properties of weaned piglets raised in a high-temperature condition [13]. Thus, *Laminaria japonica* polysaccharides with these bioactivities may positively affect the digestive tract and productivity of monogastric animals, which is of great importance for pig production.

In the current study, we hypothesized that the addition of *Laminaria japonica* polysaccharides to the diet could improve the metabolism of weaned piglets by increasing the activity of enzymes in the faeces and the growth performance of weaned piglets. Therefore, the aim of this experiment was to investigate the effects of adding *Laminaria japonica* polysaccharides to the diet on growth performance, faecal digestive enzyme activity, serum biochemical indices, and free amino acids in weaned piglets.

## 2. Materials and Methods

### 2.1. Animals and Experimental Design

The trial was conducted at the Shicheng Shanxia Pig Farm, situated in Ganzhou, Jiangxi. A total of 120 healthy crossbred barrows (Barkshire × Licha Black) were chosen for this study. The animals were selected to ensure uniformity in their initial body weights (BW, 6.13 ± 0.16 kg) and ages (21 ± 1 day). All piglets were randomly assigned to four treatments according to the initial body weight, and each concentration of LJP (0, 100, 200, or 400 mg/kg) was supplemented to the basal diet. The LJP (containing 80% fucoidan) was purchased from Xi’an Zhenlu Biotechnology Co. Ltd. (Xi’an, Shaanxi, China). Each treatment had 5 replicates (pens), each replicate had 6 piglets, and the period of the trial spanned 21 days.

The formulation of the piglets’ basal diet was produced in accordance with the nutritional prerequisites outlined by the National Research Council (2012). The detailed composition and nutritional profiles of the basal diet are presented in Table 1. The trial was conducted in a completely enclosed nursery building, where all the piglets were housed in the same room and received the feed and water ad libitum. The initial temperature in the room was kept at 28 °C and was gradually decreased by 1 °C weekly until reaching the final temperature of 25 °C. Routine inspections of the room were carried out daily, and periodic cleaning routines were implemented to maintain hygiene.

### 2.2. Sample Collection

At the end of the experiment, a single piglet from each pen, with a body weight closely aligned with the pen’s average, was selected. Blood samples were obtained via the jugular vein of these selected piglets and immediately collected into heparin tubes, allowed to rest for a duration of 2 h, and subsequently subjected to centrifuge at 3000 revolutions per minute for 10 min. Serum samples were gently extracted from the supernatant and preserved at a temperature of −20 °C for subsequent analysis. Then, faecal samples from the same piglets were collected utilizing a rectal massage. These collected samples were frozen at a temperature of −80 °C to ensure their preservation for subsequent analysis.

### 2.3. Growth Performance

At the beginning of the trial (day 0), the body weight of each individual piglet was recorded. On day 21, the pen weight of the piglets was recorded, concomitant with the quantification of the remaining feed. Concurrently, the feed consumption from days 0 to 21 was calculated. Utilizing the recorded data, the average daily gain (ADG), average daily feed intake (ADFI), and gain-to-feed (G:F) ratio were computed for each replicate. All culled or dead piglets were also recorded daily to correct the BW, ADG, and ADFI accordingly.

### 2.4. Determination of Enzymatic Activity in Faeces

Indicators of enzyme activity in faeces were measured mainly for lipase and α-amylase, using commercial kits (Nanjing Jiancheng Institute of Bioengineering, Nanjing, China). A certain mass of faeces was accurately weighed, and saline was added in a certain proportion before being gently homogenized at 4 °C, 2500 rpm, and centrifuged for 10 min. The supernatant was then diluted with saline to different concentrations, and the enzyme activities were determined according to the instructions of the kits. Lipase activity was determined using the turbidimetric method with the absorbance recorded at 420 nm; meanwhile, alpha-amylase activity was determined by the reaction of starch with iodine to form a blue complex, with a change in absorbance at 660 nm.

### 2.5. Determination of Serum Amino Acid Content and Blood Ammonia

Serum concentrations of free amino acids (AAs) and blood ammonia (NH_3_) were analysed using an automated amino acid analyser (Hitachi L-8900, Tokyo, Japan). The results of the amino acids were expressed in μg/mL, and the results of blood ammonia were expressed in mmol/L.

### 2.6. Determination of Serum Biochemical Indicators

Serum biochemical indicators encompassing glucose (GLU), total cholesterol (TC), triglycerides (TGs), high-density lipoprotein cholesterol (HDL-C), low-density lipoprotein cholesterol (LDL-C), and urea nitrogen (BUN), which were assayed using commercial assay kits (Nanjing Jiancheng Institute of Bioengineering, Nanjing, China). All procedures strictly followed the prescribed instructions by the manufacturer. Briefly, GLU levels were quantified utilizing the glucose oxidase method, wherein the alterations in absorbance were meticulously recorded at a wavelength of 505 nm. TC content was determined, employing the COD-PAP method with absorbance at 412 nm. Similarly, TG content was assessed using the GPO-PAP method, with absorbance changes recorded at a wavelength of 510 nm. HDL-C concentration was determined using the direct method, recording an absorbance of 546 nm. The level of LDL-C was measured using the direct method, recording absorbance at 546 nm. The level of BUN was measured using the diacetoxime colourimetric method with an absorbance of 520 nm.

### 2.7. Statistical Analysis

Data were analysed using one-way ANOVA using the GLM procedure in SPSS software (SPSS 27.0 version, SPSS Inc., Chicago, IL, USA). In this model, the pen was used as the unit of growth performance, while the individual piglet was the experimental unit for serum indices. Multiple comparisons were performed using Tukey HSD test. In addition, an orthogonal contrast analysis was performed to test the linear and quadratic effect among the means. Statistically significant differences were identified for values with *p* ≤ 0.05, whereas a range of 0.05 < *p* ≤ 0.10 was considered to have a tendency.

## 3. Results

### 3.1. Growth Performance

The effect of dietary LJP on the growth performance of weaned piglets is presented in Table 2. Compared with the control group, dietary LJP at dosages of 200 and 400 mg/kg exhibited a significant enhancement in the average daily gain (ADG, *p* = 0.007) and average daily feed intake (ADFI, *p* = 0.002) of weaned piglets during days 0–21, while no significant differences were observed with the addition of a 100 mg/kg dose of LJP (*p* > 0.05). In addition, LPS linearly increased ADG and ADFI (*p* = 0.002; *p* < 0.001) in piglets as the concentration of LJP in the diet increased. Nevertheless, no statistically significant variance was observed in body weight and the gain-to-feed ratio among all the treatment groups (*p* > 0.05).

### 3.2. Faecal Enzyme Activity

The effect of dietary LJP on the faecal enzyme activity of weaned piglets is shown in Table 3. Compared with the control group, the supplementation of 200 and 400 mg/kg LJP to the diet significantly increased the faecal amylase activity of piglets (*p* < 0.001), and no statistically significant differences were observed in the treatment groups with the 100 mg/kg dose of LJP (*p* > 0.05). In addition, dietary LJP linearly increased faecal amylase and lipase activities (*p* = 0.001; *p* = 0.037) and had a tendency to quadratically increase faecal amylase activity (*p* = 0.063). Moreover, when supplemented with LJP at 200 and 400 mg/kg, faecal lipase activity was numerically increased compared to the control group; however, the difference was not significant (*p* > 0.05).

### 3.3. Amino Acid Content in Serum

The effect of dietary LJP on the serum amino acid content of weaned piglets is shown in Table 4. Dietary LJP at 400 mg/kg increased serum histidine content compared to the other groups (*p* = 0.002), and dietary LJP linearly increased the contents of serum histidine and asparagine in piglets (*p* < 0.001; *p* = 0.046). In addition, dietary LJP at 200 mg/kg tended to reduce serum methionine and phenylalanine contents compared to piglets fed with LJP at 100 mg/kg (*p* = 0.075 and 0.076, respectively).

### 3.4. Serum Biochemical Parameters

The effect of dietary LJP on the serum biochemical parameters of weaned piglets is shown in Table 5. The effects of dietary LPS at 100, 200, and 400 mg/kg on the serum levels of the GLU, TC, TG, LDL-C, HDL-C, BUN, and NH_3_ of weaned piglets did not differ significantly (*p* > 0.05) compared to the control group.

## 4. Discussion

Starch is the main digestible carbohydrate component of most monogastric mammalian diets and is the main energy provider [14]. Digestion is achieved by the action of enzymes, which are secreted endogenously or through resident microbial communities. The only carbohydrate enzymes secreted by non-ruminants are salivary and pancreatic α-amylase, which breaks the α-1,4-glucosidic bond in starch [15,16,17]. However, the lack of digestive enzyme secretion in piglets after weaning causes a decrease in digestion and absorption capacities, which results in insufficient energy intake, and, eventually, inhibits the growth potential of weaned piglets. In addition, piglets develop dyspepsia and diarrhoea 1–2 weeks post-weaning [18] due to the fact that lipase and trypsin activity in the intestine increases exponentially each week from 1 to 28 days of age; however, digestive enzyme activity decreases to one-third of the pre-weaning level one week post-weaning and takes two weeks to return to or exceed the pre-weaning level [19]. As a result, piglets utilize fewer nutrients for carbohydrates and fats in non-dairy feeds during the first week of the transition from milk to solid feed. At this stage, piglets are not able to digest the proteins present in plant-based diets well or adapt to solid diets, resulting in reduced feed intake. In our study, although *Laminaria japonica* polysaccharides did not have a significant effect on lipase activity in faeces, dietary *Laminaria japonica* polysaccharides linearly increased lipase activity as the dose increased, suggesting that *Laminaria japonica* polysaccharides might have the potential to increase lipase activity. However, *Laminaria japonica* polysaccharides have been less studied in terms of digestive enzyme activities; *Lycium barbarum* polysaccharides and *Astragalus membranaceus* are also naturally occurring, plant-derived polysaccharides, and Wu et al. (2018) supplemented *Astragalus* polysaccharides in diets to improve the growth performance of young broilers, which may be attributed to the enhancement of the activity of digestive enzymes (amylase, lipase, and protease) [20], and Long et al. (2020) found that the addition of *Lycium barbarum* polysaccharides in the diets likewise improved the growth performance and the overall activity of intestinal digestive enzymes of broilers [21], which is similar to the results of the present study. In the present study, the addition of 200 and 400 mg/kg of *Laminaria japonica* polysaccharides to the diet significantly increased amylase activity in the faeces of weaned piglets, probably because *Laminaria japonica* polysaccharides also have prebiotic activity, which helps to promote starch digestion and absorption.

The dietary effect on digestive enzyme activities in the faeces was verified by the improved growth performance of weaned piglets. Che et al. (2016) reported that dietary probiotics increased the cellulase, amylase, protease, and hemicellulase activities in the faeces of weaned piglets, accompanied by an improvement in the growth performance, which might be due to the improved digestion and bone accretion, thus, having a positive effect on animal growth [22]. Walsh et al. (2012) found that the addition of *Laminaria japonica* polysaccharides to the diet increased ADG and the G:F ratio in weaned piglets throughout the experimental period (days 0–35) [23]. Heim et al. (2014) had a similar experimental period to Walsh’s, and *Laminaria japonica* polysaccharides during days 0–32 after weaning increased the average daily weight gain and weight gain to feed ratio, which were similar to zinc oxide diets [24]. McDonnell et al. (2010) also found that the addition of *Laminaria japonica* polysaccharides increased the average daily weight gain of piglets during days 0–21 post-weaning compared to basal diets [25]; this is consistent with the observations in our experiment, and we found similar results that the addition of 200 and 400 mg/kg of *Laminaria japonica* polysaccharides to the diet significantly increased the ADG and ADFI of weaned piglets, in accordance with the observation of our previous study [13], indicating that *Laminaria japonica* polysaccharides had a positive effect on piglet growth performance, which may be attributed to the improved appetite and energy absorption of piglets by the *Laminaria japonica* polysaccharides supplementation. However, there were no significant differences in body weight and G:F ratio among all treatment groups, which may be due to the short duration of the trial. Further studies are needed to determine the long-term effects of *Laminaria japonica* polysaccharides on these parameters.

It has been shown that piglets weaned in an unhealthy status could lead to intestinal dysfunction and eventually physiological disorders [26,27]. The composition of the diet has a large impact on piglets. The starch component comprises the largest portion of the diet [27]. Starch is considered a mixture of straight-chain and branched-chain starches, and highly resistant starch levels are associated with high levels of straight-chain starch [28]. The slow digestion of straight-chain starch in the gut increases the viscosity of the digesta [29], and the increased viscosity of the digesta reduces the contact of digestive enzymes with nutrients, thus decreasing the digestibility of intestinal nutrients [30,31,32], such as proteins and amino acids. Research has elucidated the pivotal role of the dietary starch digestion rate in shaping the amino acid body cycle among weaned piglets [33]. In this study, the elevated faecal amylase activity, along with the increased serum amino acid levels, may be attributed to the increased starch digestibility in the gut, which indirectly promotes protein digestion and absorption, suggesting that LJP may have the potential to improve piglet intestinal digestion of starch and positively affect protein metabolism. Furthermore, muscle protein synthesis exhibits a high sensitivity to the circulating levels of amino acids in young pigs [34,35] through the mammalian target of rapamycin (mTOR) and other signalling pathways [36,37]. Higher levels of serum AA concentrations would promote increased protein and thus growth performance in early-weaned pigs. However, there were no significant changes in the serum biochemical indices and blood ammonia levels of weaned piglets in this study, which is probably due to the short experimental period and the lack of challenge. Thus, further experiments are needed to verify the dietary effect of *Laminaria japonica* polysaccharides on serum biochemical parameters and blood ammonia levels extending the feeding period or under the challenge condition. Although the research on *Laminaria japonica* polysaccharides is being further developed, the precise regulatory mechanisms behind the observed improvements remain unclear. Further investigations are necessary to demonstrate the specific mechanisms and pathways through which *Laminaria japonica* polysaccharides influence growth and nutrient metabolism in weaned piglets.

## 5. Conclusions

The results in this experiment showed that the supplementation of 200 and 400 mg/kg of *Laminaria japonica* polysaccharides to the feed could increase growth performance and the amylase activity in the faeces of weaned piglets, which is expected to improve the nutrient metabolism ability of weaned piglets. In addition, *Laminaria japonica* polysaccharides have the potential to modulate the serum levels of amino acids.

## Figures and Tables

**Table 1:** Ingredient and calculated nutrient composition of the basal diet (as fed basis, %).

Items	Composition
Ingredients	
Expanded corn	57.50
Expanded soybean	12.00
Soybean meal	8.00
Whey powder	8.00
Lactose	8.00
Fish meal	3.00
Limestone	0.70
Calcium hydrogen phosphate	1.50
Salt	0.30
Premix ^1^	1.00
Analysed nutrient content, %	
Crude protein	20.50
Calcium	0.94
Phosphorus	0.75
Calculated nutrient content, %	
ME, MJ/kg	14.30
Lysine	1.45
Methionine	0.45

^1^ The premix provides the following per kilogram of diet: VA l5000 U, VD_3_ 2000 U, VE 30.00 IU, VK 3.75 mg, VB_1_ 2.50 mg, VB_2_ 7.00 mg, VB_6_ 2.4 mg, VB_12_ 0.03 mg, nicotinic acid 40.00 mg, calcium pantothenate 25.00 mg, folic acid 25.00 mg, biotin 0.25 mg, choline chloride 600 mg, Cu 20 mg, Fe l20 mg, Zn l60 mg, Mn 100 mg, I 0.54 mg, Se 0.27 mg.

**Table 2:** Effect of dietary LJP on growth performance of weaned piglets.

Items	LJP, mg/kg					*p*-Value		
	0	100	200	400	SEM	ANOVA	Linear ^1^	Quadratic ^1^
Body weight, lg								
Day 0	6.13	6.13	6.13	6.13	0.35	1.000	0.998	0.999
Day 21	9.31	9.72	10.28	10.32	0.44	0.340	0.109	0.444
ADG, g/d	151 ^b^	171 ^ab^	197 ^a^	200 ^a^	9	0.007	0.002	0.103
ADFI, g/d	254 ^b^	282 ^ab^	306 ^a^	318 ^a^	10	0.002	<0.001	0.114
G:F ratio	0.59	0.60	0.64	0.63	0.02	0.544	0.329	0.399

^a, b^ Means listed in the same row with different superscripts are significantly different (*p* ≤ 0.05). ^1^ Utilization of orthogonal polynomial contrast was employed to ascertain the influence of different dietary LJP concentrations.

**Table 3:** Effect of dietary LJP on faecal enzyme activity of weaned piglets.

Items	LJP, mg/kg					*p*-Value		
	0	100	200	400	SEM	ANOVA	Linear ^1^	Quadratic ^1^
Amylase activity, U/mg	45.02 ^b^	44.41 ^b^	55.83 ^a^	53.10 ^a^	1.60	<0.001	0.001	0.063
Lipase activity, U/mg	5.27	4.19	7.50	10.45	1.89	0.146	0.037	0.613

^a, b^ Means listed in the same row with different superscripts are significantly different (*p* ≤ 0.05). ^1^ Utilization of orthogonal polynomial contrast was employed to ascertain the influence of varying dietary LJP concentrations.

**Table 4:** Effect of dietary LJP on serum amino acid content (μg/mL) of weaned piglets.

Items	LJP, mg/kg					*p*-Value		
	0	100	200	400	SEM	ANOVA	Linear ^1^	Quadratic ^1^
EAA								
Histidine	12.99 ^b^	13.21 ^b^	14.47 ^b^	17.50 ^a^	0.73	0.002	<0.001	0.299
Isoleucine	12.82	15.46	12.74	14.08	1.20	0.375	0.821	0.812
Leucine	25.85	26.54	26.62	26.45	2.08	0.993	0.868	0.822
Lysine	39.83	48.38	53.31	53.19	4.61	0.223	0.084	0.239
Methionine	6.75 ^xy^	10.65 ^x^	6.64 ^y^	7.62 ^xy^	1.07	0.075	0.766	0.470
Phenylalanine	22.61 ^xy^	26.33 ^x^	21.28 ^y^	22.87 ^xy^	1.29	0.076	0.519	0.856
Threonine	24.26	32.35	27.77	26.02	3.65	0.555	0.921	0.325
Valine	27.71	30.07	29.35	31.61	2.00	0.633	0.253	0.908
NEAA								
Alanine	87.72	88.76	97.90	92.89	5.77	0.605	0.448	0.420
Arginine	15.36	9.55	9.56	16.72	3.57	0.439	0.610	0.132
Asparagine	11.95	14.51	14.58	16.30	1.27	0.187	0.046	0.572
Glutamic acid	113	133	130	128	11	0.698	0.535	0.395
Glycine	102	111	118	117	7	0.423	0.179	0.342
Proline	22.24	28.77	27.57	25.56	4.93	0.822	0.802	0.429
Serine	22.92	28.28	27.90	24.39	2.32	0.397	0.945	0.110
Tyrosine	18.21	18.88	12.95	15.30	1.80	0.132	0.148	0.299

^a, b^ Means listed in the same row with different superscripts are significantly different (*p* ≤ 0.05). ^x, y^ Means listed in the same row with different superscripts are intended to be different (0.05 < *p* ≤ 0.10). ^1^ Utilization of orthogonal polynomial contrast was employed to ascertain the influence of varying dietary LJP concentrations.

**Table 5:** Effect of dietary LJP on serum biochemical parameters of weaned piglets.

Items	LJP, mg/kg					*p*-Value		
	0	100	200	400	SEM	ANOVA	Linear ^1^	Quadratic ^1^
GLU, mmol/L	4.00	4.41	4.68	4.61	0.30	0.492	0.228	0.346
TC, mmol/L	2.45	2.43	2.29	2.48	0.15	0.830	0.922	0.436
TG, mmol/L	0.57	0.80	0.74	0.66	0.12	0.553	0.838	0.226
LDL-C, mmol/L	1.03	0.99	0.95	0.88	0.15	0.908	0.472	0.989
HDL-C, mmol/L	1.04	1.49	1.21	1.28	0.14	0.255	0.606	0.313
BUN, mmol/L	2.18	2.65	2.47	2.33	0.31	0.780	0.964	0.414
NH_3_, mmol/L	47.66	33.92	41.16	47.33	8.84	0.707	0.779	0.381

GLU = glucose, TC = total cholesterol, TG = triglycerides, HDL-C = high-density lipoprotein cholesterol, LDL-C = low-density lipoprotein cholesterol, BUN = urea nitrogen, NH_3_ = blood ammonia. ^1^ Utilization of orthogonal polynomial contrast was employed to ascertain the influence of varying dietary LJP concentrations.

## Data Availability

The data presented in this study are available upon request from the corresponding authors.
